# Attitudes Toward Pregnancy and Their Impact on Timing of Prenatal Care Initiation after Prior Spontaneous Preterm Delivery: A Qualitative Descriptive Study

**DOI:** 10.1089/whr.2024.0129

**Published:** 2024-12-09

**Authors:** Siera R. Lunn, Adwoa A. Baffoe-Bonnie, Carrie B. Dombeck, Teresa Swezey, Amy Corneli, Kelley E. Massengale, Sarahn M. Wheeler

**Affiliations:** ^1^ Duke University School of Medicine, Durham, North Carolina, USA.; ^2^ Department of Population Health Sciences, Duke University School of Medicine, Durham, North Carolina, USA.; ^3^ National Diaper Bank Network, New Haven, Connecticut, USA.; ^4^ Diaper Bank of North Carolina, Durham, North Carolina, USA.; ^5^ Department of Obstetrics and Gynecology, Duke University School of Medicine, Durham, North Carolina, USA.

**Keywords:** NH Black, NH White, prenatal care, late prenatal care, preterm birth, pregnancy attitudes

## Abstract

**Introduction:**

Timely presentation to prenatal care (PNC) is especially important for patients with a history of spontaneous preterm birth (sPTB). Our objective was to identify factors that pregnant individuals with prior sPTB perceived affected the timing of initiating PNC.

**Materials and Methods:**

We conducted in-depth interviews (IDIs) with non-Hispanic (NH) Black or NH White pregnant individuals who had a prior sPTB and presented early (<20 weeks gestation) or late (≥20 weeks gestation) to care in the index pregnancy. The IDIs focused on how patients’ initial emotions about their pregnancy and their history of sPTB(s) impacted their initiation of PNC. IDIs were analyzed via applied thematic analysis.

**Results:**

We interviewed 41 individuals (28 early presenters and 13 late presenters). Nearly all early presenters and about half of the late presenters expressed feeling both positive emotions (e.g., excitement or happiness) and worry about their pregnancies. Participants were worried about having another sPTB or miscarriage or concerned about their baby’s health. A few participants in both groups described intentionally delaying seeking care because they were worried. Also, a few early and late presenters felt uncertain about continuing their pregnancies. For a few late presenters, contemplating abortion or adoption delayed their PNC initiation; however, most participants (24 early, 4 late) sought PNC as soon as they learned they were pregnant.

**Conclusions:**

Positive emotions, worry, and uncertainty about pregnancy may influence timing of PNC initiation. These findings may inform postpartum counseling to improve the rate of patients with prior sPTB that present early during future pregnancies.

## Introduction

Prenatal care (PNC) is beneficial for the prevention of adverse maternal and infant health outcomes.^1–3^ PNC also offers accurate pregnancy-related information, timely detection of maternal and fetal complications, regular monitoring, and resources for fostering mental, social, and emotional well-being.^1,4^ Despite the numerous benefits of PNC, approximately 12.5% of pregnant people receive inadequate care, which the Centers for Disease Control and Prevention defines as initiating care after the fourth month of pregnancy or attending less than 50% of the recommended number of visits.^5^ Inadequate care is associated with a higher risk of preterm labor compared with adequate care.^6^ Therefore, prompt initiation of PNC is particularly crucial for individuals with a prior history of spontaneous preterm birth (sPTB), given their high risk of recurrence.^7^

To reduce the risk of recurrent sPTB, screenings such as serial cervix length monitoring, along with therapeutic interventions like cervical cerclage placement, may be recommended.^8,9^ These sPTB preventions are typically initiated prior to 24 weeks gestation, limiting the interventions available for pregnant individuals who present for PNC beyond that time frame.^7^ Thus, to ensure that individuals with a history of sPTB can fully access any indicated screening or therapeutics, it is imperative that care is initiated early in pregnancy.

Previous epidemiological studies have investigated barriers to prompt PNC.^10,11^ However, there are limited data from the perspective of pregnant people with a prior sPTB on how their feelings about their pregnancy influenced presentation to care. Our objective was to address this gap by exploring the attitudes of both early presenters (<20 weeks gestation) and late presenters (≥20 weeks gestation) toward their pregnancies at the time they learned they were pregnant and decision-making around PNC initiation. Understanding how initial emotions surrounding pregnancy impact when individuals with a history of sPTB initiate PNC may inform counseling in the postpartum period immediately after an sPTB. This, in turn, could contribute to improving the rate of early PNC presentation within this specific patient population during future pregnancies.

## Materials and Methods

### Parent study

The current study is a sub-study of a larger parent qualitative descriptive study.^12–14^ Through in-depth interviews (IDIs) with obstetric patients with a history of sPTB, the parent study focused on elucidating barriers and facilitators to initiating PNC. The parent study was approved by the Duke Health Institutional Review Board (Pro00100090). Written consent was obtained from all participants before participation. The semi-structured interview guide covered topics such as how and when patients learned of their pregnancies, their feelings upon pregnancy awareness, the decision-making process for when to seek PNC, and their knowledge regarding preventive measures for sPTB (Table 1). The current analysis descriptively compares results from IDIs between early and late presenters, focusing on patients’ emotions upon learning about their pregnancy and how these emotions may have influenced their timing of PNC initiation.

**Table 1.** Interview Guide Questions Referenced in the Current Analysis

Topic	Interview question
Prior pregnancy experience	[IF previous child/children is/are alive]…I see you have had X number of children preterm, how has having a baby preterm affected your life?
	How, if at all, did this experience with your health care team influence your decision on when to seek prenatal care with your current pregnancy?
Current pregnancy	How did you find out that you were pregnant?
	How did you feel when you found out you were pregnant?
	Who was the first person you told about your pregnancy?
Scheduling the first PNC visit	How much time passed before you made any efforts to seek prenatal care?
	How did you ultimately decide it was time to see a doctor for your first prenatal visit?
	About how long after you realized you were pregnant did you call to schedule your first visit with a medical provider?
Travel to the first PNC visit	Tell me about things in your life that impacted when you started prenatal care.
Preterm birth prevention	Before your first prenatal visit (for your current pregnancy), had you ever heard about any treatments to help prevent preterm birth?
	What have you heard, if anything, about the ideal time to start treatment to prevent pre-term birth?

PNC, prenatal care.

### Eligibility and recruitment

Participants were recruited from a large academic medical institution’s high-risk obstetrical clinic during a routine prenatal visit or via phone call. All recruited patients were pregnant or within 2 months postpartum of pregnancy at high risk for sPTB due to a prior spontaneous singleton preterm delivery. Additionally, the parent study focused on disparities in sPTB rates between non-Hispanic (NH) Black and NH White patients. Thus, participation was limited to patients who self-reported their ethnicity and race as NH Black or NH White.

### Data collection and analysis

Trained qualitative interviewers conducted IDIs via phone or in the clinic space. Interviews were conducted following semi-structured interview guides and audio-recorded with participant permission and transcribed verbatim (Table 1). Using applied thematic analysis,^15^ two researchers experienced in qualitative data analysis developed the codebooks. Initially, structural codes were applied to transcripts to map participant responses to interview guide questions. Then, emergent codes were developed based on participant responses and applied to text within each structural code. Structural and emergent codes are described in more detail elsewhere.^13^ To ensure consistency between coders, inter-rater reliability procedures were conducted for 28% of interview transcripts, with differences in coding discussed until reaching a mutual consensus. Two researchers then reviewed text within each emergent code to identify patterns and themes among participant experiences, which specifically described participants’ feelings upon learning about their pregnancy and the timing of PNC initiation.

## Results

We interviewed 41 individuals with a history of sPTB who were currently pregnant or within 2 months postpartum, of whom 28 were early presenters and 13 were late presenters. Of the 28 early presenters, 16 (57%) were NH Black and 12 (43%) were NH White. Of the 13 late presenters, 10 (77%) were NH Black and 3 (23%) were NH White.

Among early presenters (*n* = 28), the median number of previous term and preterm births was 0 and 1, respectively. The average gestational age at the time of their previous sPTBs was 31 weeks and 2 days (*n* = 22). Among late presenters (*n* = 13), the median number of previous term and preterm birth was 1 and 1, respectively. The average gestational age at the time of their previous sPTBs was 32 weeks and 5 days (*n* = 9).

### Initial feelings about pregnancy

Participants most frequently described experiencing both positive emotions (e.g., excitement or happiness) and worry upon learning of their pregnancies. Illustrative quotes from IDIs are listed below, with additional quotes in tables.

### Positive emotions

Nearly all early presenters (*n* = 25) reported a positive emotion, such as excitement or happiness, in response to their pregnancy. Seven late presenters expressed similar emotions (Table 2). One late presenter said, “We were excited, yeah…We was overwhelmed. That was a wonderful moment.” Many participants in both groups (*n* = 30) stated that the father of the baby, a family member, or a loved one shared their happiness. One participant described telling her partner and commented, “He was happy…we both have that same happiness.”

**Table 2.** Participant Identified Feelings upon Learning About Their Most Recent Pregnancies After Prior Spontaneous Preterm Birth

Theme	Participant	Quote
Excitement	Early presenter	*“Yes, the excitement. I think it was more excitement than concern because I just had this reassuring feeling like everything was going to be alright, so kind of the excitement overwhelmed the concern.”*
	Late presenter	*“So, I said well, I just didn’t expect it. I thought I was done you know, but it’s okay, it was exciting news. It was shocking news, but it was exciting news.”*
Worried about having another sPTB	Early presenter	*“That is the one ping part in my brain that hits me you know, multiple times a day like, am I gonna make it at least 37 weeks...Honestly, it’s like, every time I go to the bathroom and I’m like looking at like, my underwear or something like, oh, am I spotting at all? Am I bleeding you know? It’s almost, it’s probably almost every time I go to the bathroom I think about it, yeah.”*
	Early presenter	*“Yeah, so I was like, you know, we skated by on the skin of our teeth, lucky with the first one and how likely is that to happen again. So, that was always, it was part, it wasn’t necessarily a reason that I didn’t get pregnant, but it was definitely part of the equation, a factor.”*
	Early Presenter	*“Going into preterm labor again. Will this baby make it? Is it gonna be as easy as it was with __[prior baby’s name]__? Cause when I look back you know, I thought it was very complicated at first, but there were so many babies who, you know, a baby died in our pod one time you know, some babies were there for like, a year you know, there were babies and moms there who were really, really, really going through it and we were so, so, so fortunate and just the thing like, you know, am I gonna be like one of those moms who have to have their baby there long periods of times. You know, if I had to go through the NICU thing again, would it be as successful as __[prior baby’s name]__ was. Like, is it too good to be true as who she is right now and what all she’s accomplished and what all she’s beat so, you know, just concerns like that.”*
	Late presenter	*“I want to make sure it doesn’t happen again because I’m really terrified. I don’t want to have to go through all that again, but I think I’m more prepared now if it was to happen again, mentally and physically prepared.”*
Worried about having a pregnancy loss	Early presenter	*“Whether or not I would lose it, I was always concerned about a preterm delivery factor because I had heard or read like, some article or some synopsis of a study that had said when you have an unexplained preterm delivery you are more likely to have another one and potentially just earlier and earlier.”*
	Late presenter	*“I worry about just having a miscarriage anyway. I worry about you know, my health, I worry about the baby’s health…you’re trying to do everything right, you don’t want to do nothing wrong and then just trying to hold it, you’re trying to hold on. So, that’s what I deal with every day.”*
Worried about their baby’s health	Early presenter	*“Yeah, and if he would have Trisomy 18 or any of those things or if anything would be wrong with this child or not, but so far, he’s doing good.”*
	Late presenter	*“None really. Well, I guess just whether they’re full-term or preterm it’s just if they got all their fingers. You know just the normal little things. If they can see. Actually, and I don’t know why, I’ve always wondered about if they’ll be blind or deaf; I don’t know why.”*
Uncertainty about continuing their pregnancy	Early presenter	*“Because I really didn’t really have any intentions on really keeping it at all cause I wanted to either get an abortion or I was gonna give it up for adoption cause I didn’t want it, but I grew to you know, show that motherly love, okay, understand what this happened, but me and my family, we don’t believe in abortions so you can’t do that...”*
	Late presenter	*“Like I said, at the time we weren’t planning on keeping it but then when we did make the decision to keep it I tried to call OB/GYN to see where I would be able to go to. I want to say I was like around 12 weeks at the time.”*
	Late presenter	*“...At first it was debatable whether I was going to keep it or not. It was debatable but then I was like okay time is going by. It’s too late to do anything about it; might as well go to the doctor.”*

sPTB, spontaneous preterm birth.

Due to their previous experiences with sPTB, some participants, especially within the early presenter group, explained that it was challenging to feel completely positive. Instead, they felt “cautiously optimistic.” For example, a participant stated, “Happy, but you know, still apprehensive and anxious. That first trimester’s always a harder time cause anything could happen and you don’t, it’s like you don’t want to get, you’re excited but you don’t want to get too excited, but overall, still happy, just you know, cautious, I guess.”

### Worry

Worry was the second most frequently reported feeling for participants in both the early (*n* = 24) and late presenter (*n* = 6) groups. Participants described being worried about experiencing another sPTB (early: *n* = 13; late: *n* = 5) or pregnancy loss (early: *n* = 10; late: *n* = 1). Both early (*n* = 5) and late (*n* = 3) presenters also expressed concern about their baby’s health.

#### Worried about having another sPTB

The majority of early and late presenters who voiced that they worried about their pregnancy expressed concern about having another sPTB. It was the most common worry among both groups. For example, an early presenter described knowing that her history of sPTB increased her risk of recurrence. Another early presenter described how every time she went to the bathroom, she checked for bleeding and wondered if she would reach 37 weeks. She stated, “That is the one ping part in my brain that hits me you know, multiple times a day like, am I gonna make it at least 37 weeks…Honestly, it’s like, every time I go to the bathroom…oh, am I spotting at all? Am I bleeding you know…” A late presenter mentioned that she was terrified about the prospect of having another sPTB but felt that she was better equipped to navigate the situation due to her previous experience. Additionally, two early presenters questioned whether they had gotten lucky with their initial experience of sPTB and whether they would be as fortunate if it happened again.

#### Worried about having a pregnancy loss

More early presenters (*n* = 10) described being concerned about experiencing a pregnancy loss than late presenters (*n* = 1). Nearly all early presenters who expressed worry about a recurrent loss mentioned that they previously suffered a miscarriage, tubal pregnancy, or neonatal death. One early presenter described the difficulty of not having a definitive reason for why her pregnancy loss occurred. She commented,

*The concerns really were the unknown. I was expecting for the doctors to tell me okay, you lost your baby, or you went into labor because of this and because of that, but when I came in and Dr. _______ just looked at me and shook her head and said, I can’t tell you anything you know, your case is different and it’s just odd for you to have five normal pregnancies with no complications and then you get to number six and you have a complication…So, I was afraid to go on that ride again especially because it was unknown of why it happened…*

Another participant who presented early to PNC mentioned that she had to forgive herself because she initially believed that her pregnancy loss was her fault. To help prevent another pregnancy loss during her current pregnancy, she said that she was being overly cautious. Similarly, a late presenter reported that she was avoiding doing anything that could potentially harm her baby. She stated, “I just made sure I took my prenatals, made sure I didn’t do anything I wasn’t supposed to…as far as lifting or anything.”

#### Worried about their baby’s health

Patients in both the early and the late presenter groups described being concerned about negative infant health outcomes, such as prolonged neonatal intensive care unit stays, low birth weight, prematurity, or congenital anomalies. One early presenter recounted, “My first concern was thinking if I have a baby, if it’s gonna be underweight, is it gonna be full term.” Similarly, a participant that presented late to PNC commented, “Well, whether he was gonna come early. Was it gonna be premature? If he did come out premature, would it be you know, rough for him or you know, would things go by easy…”

### Uncertainty about pregnancy continuation

A few patients in both the early and late presenter groups said that they felt uncertainty about continuing their pregnancies (*n* = 2 vs. *n* = 3, respectively). The two early presenters considered abortions, but their families didn’t believe in abortions. The experience of one of the early presenters is portrayed in the following quote:

*They’re mostly like you know you have a baby you know, you keep it; don’t kill it. You know and so when I told them that I was pregnant and when I told them I was going to have an abortion they was like mad and then on me all hard and I don’t drive so like my family got to take me wherever I need to go or I got to catch the bus and when I had an appointment my aunt wouldn’t take me when she found out that I was going to kill it so basically they made me keep the baby.*

Of the three late presenters, one mentioned that she considered adoption but eventually decided to parent her baby. She stated, “I mean, and it sounds bad, but I was actually considering adoption in the beginning…” The other two late presenters contemplated abortion. After learning they were pregnant, they both waited approximately 7–8 weeks to initiate PNC due to uncertainty about keeping their pregnancies. One of those participants said that deciding to continue her pregnancy was the foremost factor that impacted her presentation to PNC.

### Timing of presentation to PNC

#### Presented promptly upon pregnancy awareness

Of the 41 total participants, 28 (early: *n* = 24; late: *n* = 4) reported promptly scheduling their initial prenatal appointment once they learned they were pregnant. Several participants commonly expressed that upon becoming pregnant, scheduling a doctor’s appointment should be the next step. For example, an early presenter said, “I just, well, I just think as soon as you get pregnant you should do prenatal care, so that’s kind of how I knew. I just figured that the earlier that they can monitor your baby and make sure everything’s okay the better it will be in the long run.” Likewise, when asked about the best time to initiate PNC, a late presenter answered, “It’s whenever as soon as you find out…When you find out you’re pregnant you need to yes have prenatal care.”

Some of the participants (early: *n* = 4; late: *n* = 1) who promptly scheduled a PNC appointment discussed that they initiated care quickly because of adverse experiences during their previous pregnancies and concern about recurrence (Table 3). They explained that presenting promptly to PNC enabled them to make sure everything was okay. An early presenter said, “Oh, right away. As soon as the pregnancy test popped up positive, I was on that phone cause I wanted to get the earliest as possible prenatal care as soon as possible…Because of the last pregnancy in 2017. I didn’t want nothing, you know, to go wrong.”

**Table 3.** Participant Identified Feelings That Impacted the Timing of Prenatal Care Initiation After Prior Spontaneous Preterm Birth

Timing	Participant	Quote
ASAP	Early presenter	*“Yes, and previous to that so in 2017, I had a miscarriage…So that’s why I kind of tried to use that as a reason to get to see them early. So, I was like you know I’ve had a miscarriage in the past…And it was like a late-term miscarriage so I’m like I just want to make sure like everything is okay...”*
	Early presenter	*“Well, of course you’re more cautious when things don’t go normal. Maybe if I didn’t have a preterm and lose a baby, I probably would have waited because I’m like, I got this, I know what to do and I know what to look for, but because I knew that I was high risk then I needed to seek care as soon as possible, so that if there’s anything that could be prevented from the beginning then I could do it early on versus waiting, so.” *
	Late presenter	*“I mean, after I found out I was pregnant I knew I needed to get the prenatal care you know…I definitely have to get prenatal care because I can’t afford for nothing to* *go wrong.” *
Intentionally delayed care	Early presenter	*“…I you know, I think that I definitely wanted to wait a week, but that is, you know, a lot of miscarriages happen before six weeks, so let’s wait a week to even call the doctor… I still have that thought of more so of having the miscarriage for this pregnancy.” *
	Late presenter	*“Well, because I had my first child early, I guess it was traumatizing you know, to go through all that so soon, so young…I just wanted to make sure it was gonna be healthy, it was gonna be full term, so I tried to wait it out. I had my first at 24 weeks, so I wanted to try to wait it out and be like, oh well, if it makes it past 24 weeks, I’ll feel a lot better you know, so I pretty much stretched my PNC and was scared to get a bad report or come in and hear something negative so I stretched it out as long as I could really, by not having it.”*
No PNC	Late presenter	*“What happened was I had called Durham and to make an appointment and they couldn’t see me because I was further along than 16 weeks. So then I had to go through someone else to get it and it was just a battle with Medicaid trying to get my card, the insurance, because they were trying to say I still didn’t have it when I got my card.”*
	Late presenter	*“31 weeks; when I was able to get to the health department… Well to try and get to the health department was kind of hard, so. Especially with that trying to confirm the whole pregnancy thing and two people first come, first serve. Yes, that was kind of hard to do.”*
Fatalistic	Early presenter	*“No, I felt like it’s just what I do now. So, it’s like I just know that they’re just going to be preterm and I’m okay with that because, maybe because the other ones were healthy.” *
	Late presenter	*“It wouldn’t matter because I found out I was two weeks pregnant with my oldest and I still had it.”*

PNC, prenatal care.

The four late presenters who promptly initiated PNC upon discovering their pregnancies reported late pregnancy awareness. They described learning about their pregnancies when they were near or beyond 20 weeks gestation. One participant recounted, “So when I took sick and went to the hospital, I found out I was pregnant with this one. Well, I was already about four or five months.” Another participant stated, “I started coming here ever since I was like 20 weeks, when I found out I was pregnant.”

#### Intentionally delayed care

A few participants (early: *n* = 2; late: *n* = 2) described intentionally delaying care because they were worried about adverse outcomes, including sPTB and miscarriage. One late presenter commented, “…Actually I think it did made me come later because I was like scared and terrified what might happen this time even though it probably wasn’t the best decision.” Another participant believed that if they waited to initiate PNC until 24 weeks, the gestational age of their previous delivery, they might achieve a better outcome. They viewed PNC as an opportunity to receive bad news and wanted to avoid that.

### No PNC

At the time of their IDI, two late presenters had not received any PNC, although one participant had her initial prenatal appointment on the same day of her IDI. Both reported insurance status and scheduling difficulties as barriers to initiating care. For example, one of them mentioned that delays in Medicaid approval and receiving her insurance card prevented early PNC initiation. Although one participant explained that she did not receive PNC due to appointment times conflicting with her work schedule, she had family and friends in the health field who would provide advice or monitoring when needed. She recounted her experience:

*…I could never find a good way around my schedule so, to do a prenatal appointment exactly, so this pregnancy I didn’t have prenatal care at all. My aunt is a registered nurse though, and I know my friend, her grandmother is a pediatrician…They made it all, so if I ever felt like, you know, it was mandatory, I went to her (aunt) most definitely and she would tell me whether or not I needed to go in or whether things was normal.*

### Fatalistic

Three participants (early: *n* = 1; late: *n* = 2) said they believed they would have negative outcomes regardless of when they presented for PNC. For example, one late presenter commented:

*When I went, I started on time the last time and I’m like, okay, I went and did everything, all the precautionaries I could take, and did all this, and this still happened so if I go later it wouldn’t make a difference if something was to happen because whatever is going to happen is going to happen, regardless…*

One late presenter stated that the timing of PNC initiation is inconsequential, citing her prior experience of still having an sPTB despite learning she was pregnant at two weeks and promptly seeking care. One participant that presented early believed that she would always have sPTBs and was okay with that because her other children were healthy.

## Discussion

We analyzed IDIs of individuals with a history of sPTB about their feelings upon pregnancy awareness and the factors that influenced their timing of PNC initiation in a subsequent pregnancy. Participants in both the early and late presenter groups most commonly felt positive emotions and worry. Worry about adverse pregnancy outcomes facilitated early and late presentation in our cohort. Additionally, a few early and late presenters felt uncertainty about continuing their pregnancies. For late presenters, contemplating abortion or adoption delayed their PNC initiation by weeks. Most IDI participants presented promptly to PNC upon pregnancy awareness, including four late presenters who reported late pregnancy awareness.

Previous research has shown that having a positive attitude about pregnancy can encourage prompt presentation to PNC.^10,11^ Daniels et al. examined how attitudes toward pregnancy affected the timing of PNC initiation among a cohort of Black women of low socioeconomic status and found that early presenters commonly reported feeling excited about their pregnancy, whereas late presenters generally felt indifferent or devastated.^11^ Although the late presenters in our cohort did not express indifference or devastation, more early presenters felt positive emotions upon learning of their pregnancy compared to late presenters. Notably, 75% of the late presenters in our cohort who promptly initiated PNC voice that they felt excited about their pregnancy. The difference in our findings compared with Daniels et al. may be due to different patient populations and the definition of early versus late presentation. We recruited pregnant people with a prior sPTB from a high-risk obstetrical clinic, whereas Daniels et al. focused on Black women who received PNC at community clinics. Daniels et al. defined early presentation as PNC within the first trimester, while our study defined late presentation as beyond 20 weeks gestation, given the potential implications for access to preterm birth prevention.^11^

Worry about poor pregnancy outcomes may have contributed to early and late presentation in our cohort. For some participants, PNC was an opportunity for participants to ensure that their baby was healthy. Daniels et al. identified a similar theme in their cohort of patients seeking PNC from community clinics; some participants felt fearful of potential adverse outcomes if they did not seek out PNC for regular monitoring of the pregnancy.^10,11^ On the contrary, both early and late presenters in our study expressed that worry or the fear of receiving bad news deterred them from initiating PNC ASAP. Likewise, in a cohort study of individuals who had previously suffered a perinatal loss, Cote-Arsenault et al. found that patients were terrified that prenatal tests and ultrasounds would bring disappointing news.^16^ Our findings add to existing literature by uncovering how worried or anxious emotions may impact when individuals at high risk for preterm delivery initiate PNC. Although early and late presenters experience similar emotions, these feelings may lead them to seek care at completely different times in their pregnancies. Further research is needed to investigate whether mental health support following an adverse pregnancy outcome could encourage early PNC in future pregnancies.

Some participants also expressed a fatalistic attitude, believing that they would have recurrent sPTB regardless of when they presented for PNC. This perspective appeared to cause two late presenters to delay their care. While our research and previous studies indicate that both early and late presenters understand the importance of timely PNC initiation,^11,17^ some individuals struggle to fully appreciate the need for prompt presentation when they have previously experienced pregnancy-related complications despite receiving PNC early. Future research should explore interventions that could help pregnant people with a fatalistic attitude present early to PNC despite their prior birth outcomes.

In addition to fatalistic attitudes, uncertainty about continuing their pregnancies led a few late presenters to intentionally delay PNC. Unintended pregnancy has been identified as a common barrier to early PNC initiation in prior studies.^10,11,17,18^ Our data were collected prior to the 2023 Supreme Court decision overturning *Roe v. Wade*. The current political climate may have a significant influence on when patients initiate PNC. Future research should explore the effect of this decision on timing of PNC initiation.

In light of recent changes in abortion access, it is especially important that patients are aware of their pregnancies as early as possible. Our study revealed that some late presenters only learned of their pregnancies when they were near or beyond 20 weeks gestation, hindering prompt presentation to PNC. Similarly, Krukowski et al. found that late pregnancy awareness is a barrier to early PNC initiation.^19,20^ Previous studies have also found that patients who discover their pregnancy at an advanced gestation tend to be less informed about the signs and symptoms of pregnancy.^11,18,19^ Prior research suggests that initiatives aimed at improving timely presentation to PNC should include educating patients on the range of pregnancy-related symptoms and differentiating between pregnancy symptoms and other health issues.^11,18,19^

Although the current analysis focused on participants’ attitudes and emotions, there are additional important barriers to care that were explored during the IDIs and reported in other articles.^13^ Participants who had delayed or no PNC prior to their interview identified insurance and scheduling issues as barriers to timely PNC initiation. Mazul et al.’s cohort of low-income African American women reported these same barriers to prompt presentation and others, including transportation limitations, financial strain, limited social support, and extended clinic wait times.^10^

Another study highlighted the frustrating conundrum of accessing Medicaid, where patients require Medicaid to see a provider yet need a provider’s confirmation of their pregnancy to qualify for Medicaid.^20^ It is important to note that Medicaid coverage was recently expanded in North Carolina, the state where these IDIs were conducted. Perinatal Medicaid coverage rates increased in states that approved the expansion.^21^ Thus, the recent expansion may help alleviate the barrier of insurance status, enabling patients to initiate PNC earlier. Future research should explore the impact of Medicaid coverage expansion on the timing of PNC initiation in North Carolina. Furthermore, similar to our findings, Sunil et al. and Fryer et al. both found that patients’ inability to secure a prenatal appointment on their preferred date and time contributed to delayed presentation of care.^17,22,23^ Limited appointment availability, partly due to a shortage of health care workers, emphasizes the crucial need for an expanded workforce to better meet patient needs.^22^

It is important to highlight that 77% of late presenters identified as NH Black. Consistent with our study’s themes, Wong et al. found that Black women who did not initiate PNC as early as desired reported poorer psychological well-being.^23^ This reinforces the idea that emotions may impact PNC initiation. These findings underscore the critical role that mental health interventions could play in facilitating timely presentation to PNC among Black patients.

Based on our findings and previous studies, individuals with a prior sPTB grapple with a range of emotions, which can affect subsequent pregnancies.^24^ These feelings may cause some to initiate PNC ASAP, while others decide to wait weeks. Thus, it is advisable for clinicians to counsel patients during postpartum visits following challenging or traumatic deliveries. This counseling should explore patients’ sentiments regarding their delivery and future childbearing plans and address pregnancy symptoms and the importance of early PNC. Offering anticipatory guidance on prompt PNC initiation may positively impact the timing of PNC presentation in subsequent pregnancies.

Although our study contributes valuable insight on how attitudes about pregnancy influenced presentation to care among a cohort of patients with prior sPTB, there were limitations. The IDIs were conducted in a single health care system prior to the COVID-19 pandemic and the overturning of *Roe v. Wade*; similar or different emotions could be identified from participants from other health care systems or in environments with limited abortion options. Despite these limitations, our findings add to the sparse literature that captures perspectives from patients who received late PNC. They underscore that while both early and late presenters share similar feelings about their pregnancies, the same emotions can guide them to seek care at distinctly different times.

## Conclusions

Our research suggests that both early and late presenters experience similar feelings of positive emotions, worry, and uncertainty upon pregnancy awareness, which may influence the timing of PNC initiation. These findings may inform postpartum counseling to improve the rate of patients with a history of sPTB that present early in subsequent pregnancies.

## Abbreviations Used

IDI: In-depth interview
NH: non-Hispanic
PNC: Prenatal care
sPTB: Spontaneous preterm birth
